# Predictors of Endocrine Resistance in a Cohort of Mexican Breast Cancer Patients

**DOI:** 10.21203/rs.3.rs-4414887/v1

**Published:** 2024-05-30

**Authors:** Jonathan González-Ruíz, Mary Beth Terry, Paula Cabrera-Galeana, Alberto Monroy-Chargoy, Carol Horowitz, Nina Bickel, Claudia García-Cuellar, Andrea Ramírez, Enrique Bargalló, José Diaz-Chavez, Salim Barquet-Muñoz, David Cantú-de-León, Diddier Prada

**Affiliations:** Instituto Nacional de Cancerología; Columbia University; Instituto Nacional de Cancerología; Instituto Nacional de Cancerología; Icahn School of Medicine at Mount Sinai; Icahn School of Medicine at Mount Sinai; Instituto Nacional de Cancerología; Instituto Nacional de Cancerología; Instituto Nacional de Cancerología; Instituto Nacional de Cancerología; Instituto Nacional de Cancerología; Instituto Nacional de Cancerología; Icahn School of Medicine at Mount Sinai

**Keywords:** Breast cancer, Endocrine resistance, Prognosis, Cancer treatment

## Abstract

**Purpose:**

This study aimed to determine the prevalence of endocrine resistance in a cohort of Hispanic Mexican breast cancer (BC) patients receiving care at Instituto Nacional de Cancerología (INCan). Additionally, the clinical-pathological factors associated with endocrine resistance were identified, and their impact on patient survival was explored.

**Methods:**

A retrospective analysis of 200 BC patients who attended INCan between 2012 and 2016 with estrogen receptor (ER) and progesterone receptor (PR) positive tumors was made. Endocrine resistance was defined according to the International Consensus Guidelines for Advance Breast Cancer 2 definition. Their clinicopathological characteristics were analyzed to determine the association with endocrine resistance presence. We used sensitivity analyses and multivariate-adjusted logistic regressions, Kaplan-Meier curves, and multivariate-adjusted Cox regressions. P-value < 0.05 was considered as statistically significant.

**Results:**

Endocrine resistance was observed in 32.5% of patients included in this study. The distinction between hormone resistance and sensitivity was influenced by tumor size and node status. It had a mean diameter of 7.15 cm in endocrine resistance cases compared to 5.71 cm in non-endocrine, with N3 status present in 20% of endocrine resistance cases versus only 2.2% in non-endocrine (p-value < 0.001). The clinical stage exhibited a strong association with endocrine resistance (Risk Ratio [RR] 4.39, 95% confidence interval [95%CI] 1.50, 11.43). Furthermore, endocrine resistance significantly impacted mortality during the follow-up, with a Hazard Ratio [HR] of 23.7 (95%CI 5.20, 108.42) in multivariable-adjusted models. However, a complete pathological response reduced the endocrine resistance risk, as demonstrated by a Risk Ratio (RR) of 0.15 (95% CI 0.03, 0.75).

**Conclusions:**

Advanced clinical stage at diagnosis predicted endocrine resistance in Hispanic Mexican BC patients. Complete pathologic response in locally advanced disease patients was also a key predictor of endocrine resistance. These results indicated that endocrine resistance was a critical factor in BC during follow-up.

## INTRODUCTION

Every year, ~ 250,000 new BC cases are diagnosed in the United States, with 80% being hormone receptor-positive [1]. BC is the leading cause of cancer-related mortality in Mexican women since 2006 [2]. Moreover, BC is the number one prevalent neoplasm in Mexican women (15.23%), according to The Global Cancer Observatory [3]. A retrospective study conducted at INCan analyzed 5,500 BC patients from 2007 to 2013 and indicated that 60.3% had hormone receptor-positive tumors [4].

The annual global estimate of BC-related deaths exceeds 450,000 despite reductions in BC mortality because of available treatments. The incomplete understanding of BC’s biological heterogeneity is the key contributing factor [5]. Over 761 BC samples were analyzed at DNA, RNA, and protein levels under The Cancer Genome Atlas (TCGA) Project, and identified distinct molecular types leading to four main phenotypes: Luminal A, Luminal B, epidermal growth factor receptor 2 (HER2)-enriched, and Basal-like [6]. Luminal A and Luminal B type patients have positive hormone receptor expression. Endocrine therapy in this group is thus the basis of treatment. However, resistance may occur during this treatment [7, 8], known as endocrine resistance.

Jensen and Jacobson made the seminal discovery of estrogen receptor (ER+) in 1960 [9]. This and the subsequent studies have established estrogen’s role in BC pathogenesis [10]. Estrogens modulate their activity via the two receptors, ERα and Erβ [11]. ERα is overexpressed in BC and is the key target for hormonal therapy [12]. A minimum of six therapeutic modalities dictate endocrine therapy in adjuvant and metastatic setting, namely selective endocrine resistance modulators (SERMs), selective endocrine resistance down-regulators (SERDs), aromatase inhibitors (AIs), mTORC1 inhibitors with aromatase inhibitors, cyclin-dependent kinases 4 and 6 (CDK4 and CDK6) inhibitors with AIs, and finally CDK4 / CDK6 inhibitors with SERD [8]. A study by the Early Breast Cancer Trialists Collaborative Group (EBCTCG) provided evidence that adjuvant therapy with tamoxifen at five years reduced the recurrence and death from BC by 40% and 20%, respectively [8, 13].

Endocrine resistance clinically manifests as early disease recurrence or progression to advanced stages [1, 13]. Studies have reported that 40–50% of patients show recurrence during adjuvant treatment. Most patients in metastatic settings recur within 2–3 years of endocrine therapy initiation [13]. Endocrine resistance was defined and described in 2014. Its purpose and criteria were established through the consensus of experts to provide an international description, which allowed for standardized inclusion criteria in clinical studies. These criteria were not necessarily proposed for routine clinical decision-making [13]. Primary endocrine resistance is characterized by the recurrence in the first two years of adjuvant endocrine therapy or disease progression in the first six months of first-line endocrine therapy in a metastatic setting [7]. Secondary endocrine resistance is considered when the recurrence occurs after two years of adjuvant endocrine therapy or disease progression after six months of initiating first-line endocrine therapy in a metastatic setting. The multifactorial genesis of endocrine resistance may occur through the activation of alternative oncogenic signaling pathways in tumor cells and tumor microenvironments [1]. Numerous studies have explored the molecular pathways of endocrine resistance [8]. However, the clinical and pathological factors related to endocrine resistance have not been characterized, particularly in Hispanic Mexican populations. Access to biomarkers or genomic signatures is complicated in low- or middle-income countries, so this approach can facilitate clinical and therapeutic strategies for managing BC patients in locations with no access to molecular data [14, 15].

There is a tremendous lack of information on endocrine resistance in Hispanic BC patients. This study focused on BC-diagnosed patients with positive hormone receptor expression who were treated at the Instituto Nacional de Cancerología (INCan) - Mexico between January 2012 and December 2016. Patients were grouped according to the definition of the International Consensus Guidelines for Advanced Breast Cancer 2 (ABC2) [16]. Their clinicopathological characteristics were analyzed to determine the association with endocrine resistance presence per the ABC2 criteria. Furthermore, the relationship between endocrine resistance and patients’ mortality was explored.

## MATERIALS AND METHODS

### Population

This study included BC-diagnosed patients who attended INCan between 2012 and 2016. Patients with hormone receptor-positive and HER2-negative breast cancer detected by immunohistochemistry assay were included. The INCan Institutional Review Board approved the project (IRB Approval Number: Rev/0040/20).

### Clinical data

The socio-demographic, clinical, surgical, and follow-up data were collected from the electronic medical records of each patient. A database was created with the following variables: Age at diagnosis (continuous variable), age at menarche (continuous variable), body mass index (BMI, continuous variable), tumor size (continuous variable), lymph node (presence or absence), cellular proliferation (Ki67 percentage, continuous variable), comorbidities (diabetes, hypertension, overweight, obesity), smoking (yes or no), menopause (yes or no), histological grade, histological type, histological phenotype by immunohistochemistry, clinical stage, and complete pathological response (CPR; yes or no).

The positivity of hormone receptor expression was determined by following the American Society of Clinical Oncology (ASCO)/College of American Pathologists (CAP) criteria, with a threshold of > 1% estrogen and/or positive progesterone receptor expression [17, 18]. The clinical stage was assigned according to the American Joint Committee on Cancer (AJCC) 8th criteria[19]. St. Gallen criteria was employed to classify the patients’ phenotypes[20]. Two groups were established, i.e., patients with primary or secondary endocrine resistance as per ABC2 criteria, and those classified as hormone-sensitive [16]. Follow-up involved the data gathering from records and assessing the endocrine resistance based on ABC2 criteria in the follow-up period. Recurrence was defined with the presence of new local or distant lesions.

### Statistical analysis

Descriptive techniques were employed for acerating clinical and pathological characteristics of patients. Student’s t-test or Mann-Whitney U test was used for the continuous data to determine differences between hormone resistant and hormone sensitive groups. Chi-squared test (χ2) was employed for the categorical data based on their normality. A multivariable Cox regression model was used to explore the association between endocrine resistance and mortality. Hazard ratios (HR) were calculated with 95% confidence intervals. Statistical difference was set at *p*-value < 0.05 for all the data analysis. Statistical tests were conducted, and graphs were plotted using R software (R Project for Statistical Computing, CRAN, The Comprehensive R Archive Network, Vienna, Version 4.0.2).

## RESULTS

A total of 200 cases diagnosed with hormonal receptor positive and HER2 negative BC were evaluated. Among them, 65 cases (32.5%) met the clinical criteria for endocrine resistance, while 135 cases (67.5%) were hormone sensitive. The mean age at menarche was 12.82 years with standard deviation (SD) of 1.92 years. Mean tumor size at diagnosis was 6.18 cm with SD of 3.00 cm. Clinical stage distribution revealed that 40% of endocrine resistance group had stage IV, while 43% of hormone-sensitive group had stage IIIA. Recurrence was observed in 42 patients, i.e., 21.00% of the total population. A difference in tumor size (7.15 cm, SD: 3.40 cm vs 5.71 cm, SD: 2.68 cm; *p*-value = 0.003) and clinical stage *(p*-value <0.001) was found upon comparing the endocrine resistance and hormone-sensitive groups. No significant differences were found in age, BMI, comorbidities, histological grade or type between the two groups. The description of variables’ distribution evaluated for both is depicted in Table 1. Regarding clinical stage analysis, tumor size and lymph node status differed between the endocrine resistance and hormone-sensitive groups, but not metastasis status (Table 2).

### Factors independently associated with endocrine resistance

**Figure 1:**
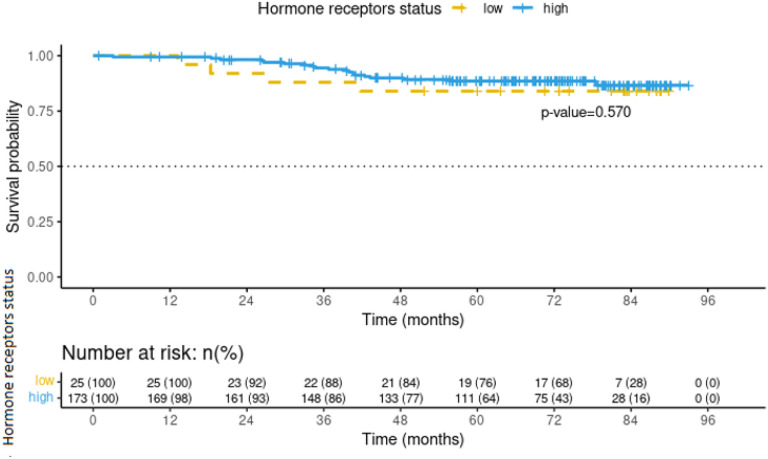
Mortality curve of breast cancer patients in relation to the intensity of hormone receptors, attended INCan. Follow-up until May 2020.

**Figure 2:**
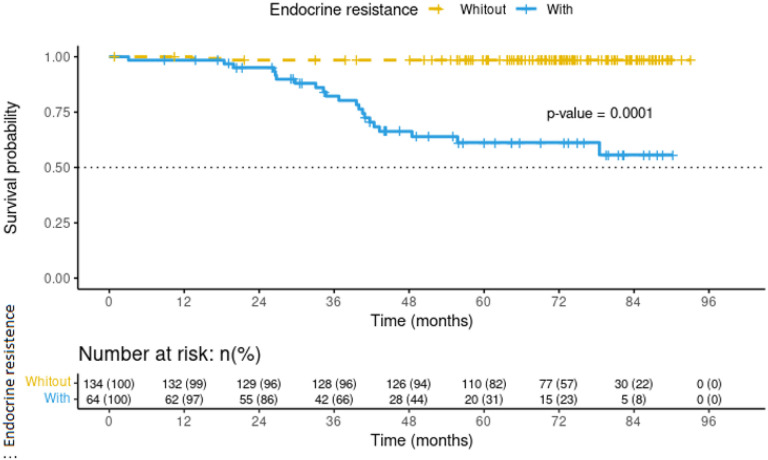
Kaplan and Meier curve for overall survival in breast cancer patients at INCan. Follow-up until May 2020.

Factors independently associated with endocrine resistance were determined by sensitivity analyses which included the age adjustments, age at menarche, Ki67, phenotype luminal A vs. luminal B, BMI, histologic subtype, and the model fitness according to Akaike Information Criteria (AIC). Clinical stage was emerged as an independent factor and associated with endocrine resistance in this population (Risk Ratio [RR] 4.14, 95% confidence interval [95% CI]: 1.525 – 11.246; p-value <0.006) (Table 3). Multivariate analysis incorporating clinical stage as covariate revealed that endocrine resistance was associated with 23-fold increase in mortality in the follow-up (HR 23.7, 95% CI: 5.20 – 108.42, *p*-value <0.001) (Table 4). No differences in mortality were observed upon analyzing the intensity of estrogen and progesterone receptors, suggesting a potential lack of association on mortality vs. low statistical power (*p*-value 0.57) (Figure 1). Survival rates were different between the two groups after a follow-up of 75 months. At the time of this analysis and among the patients without endocrine resistance, 29% were alive while 6% patients with endocrine resistance survived (p-value 0.0001) (Figure 2).

### Endocrine resistance in locally advanced disease

**Figure 3:**
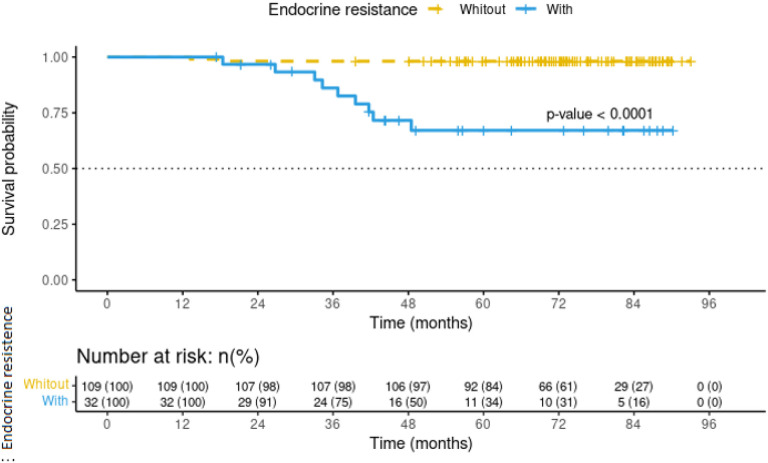
Mortality curve in locally advanced breast cancer patients treated at INCan. Follow-up until May 2020.

Clinical stage emerged as the most critical factor (RR 2.92, 95% CI: 1.20, 7.11; *p*-value 0.018) for endocrine resistance in locally advanced disease patients. However, it was also observed that the pathologic complete response was a protective factor (RR 0.15, 95% CI: 0.03, 0.75; *p*-value 0.020) against endocrine resistance (Table 5). Furthermore, endocrine resistance persisted as an independent risk factor for the mortality in this patients’ subgroup (Figure 3).

## DISCUSSION

The findings in this study indicated that clinical stage, particularly depending on tumor size and node status, was an independent predictor of endocrine resistance as defined by ABC2 criteria. This finding is consistent with previous studies that had assessed the association between clinicopathological variables suggesting that clinical stage is an independent prognostic factor among the endocrine resistant luminal BC patients, particularly those undergone modified radical mastectomy [21, 22]. We also confirmed the importance of CPR in locally advanced disease. This is the first study of its kind in Hispanic Mexican populations. No associations were identified for factors such as Ki67, BMI, histologic subtype, or phenotype (Luminal A vs. B). Furthermore, it was found that endocrine resistance was an independent factor of mortality. Our study is also one of the first suggesting endocrine resistance as an independent factor for mortality in BC patients.

Limited studies have sought a connection between patients’ clinical characteristics and the predictive value of mortality. Fewer studies have assessed the clinical characteristics of patients with endocrine resistance. Sestak et al. in 2013 evaluated the predictors of recurrence in hormone-sensitive BC patients. They underscored the nodal status and tumor size as the primary prognostic factors, which affected recurrence in 5 to 10 years. A comprehensive 20-year analysis conducted by the Early Breast Cancer Trialists’ Collaborative Group revealed that pertaining to the adjuvant therapy, tumor size, nodal status, and histological grade emerged as the key clinical variables linked with recurrence throughout the extended 5 to 20-year window [23].

The endocrine resistance had been associated with mutations in the ligand-binding domain of the *ESR1* gene [24]. However, conducting such determinations in developing countries posed challenges. In this study, it was addressed by using the clinical definition of endocrine resistance based on ABC2 criteria. This approach was effective in identifying patients with endocrine-resistant tumors and assessing their implications on mortality. It was found that the endocrine resistance was an independent factor and unaffected by other covariates.

The patients’ cohort in this study was followed over time and represented BC characteristics in Mexico in terms of mortality. Dowsett’s study evaluating the patients’ cohorts ATAC and BIG 1–98 suggested that even in the era of high-precision medicine, tumor size and lymph node status still had value as the predictors of recurrence and endocrine resistance in hormone-sensitive BC patients [25]. Determining which patients develop endocrine resistance remained challenging in resource-limited settings. Decision-making based on other variables could lead to undertreatment or overtreatment in overcoming the endocrine resistance [26]. The criteria for defining endocrine resistance had thus been correlated with long-term mortality and also validated the requirements employed by International Consensus Guidelines for ABC[27].

This study had certain limitations such as relatively smaller number of patients included compared to other BC studies. However, this sample size was robust to facilitate a comprehensive multivariate analysis adjusted for the key covariates. Additionally, because of the observational nature of this study, the determination of endocrine treatment was the discretion of individual treating physician. This resulted in variations regarding the treatment approaches and heterogeneity for potential therapeutic responses analyses.

This is the first study in Latino population of Mexican women which depicts the significance of advanced clinical staging at the time of breast cancer diagnosis. This offers a straightforward and accessible method for low- and middle-income country to identify high-risk population and empower clinicians to focus on those at the greatest recurrence risk. Moreover, this allows the selective targeting of patients to take benefit from innovative drugs for combating endocrine resistance. It is identified that tumor size in combination with nodal status remains the most important predictor of endocrine resistance in patients undergoing endocrine therapy. The decision making based on other variables may lead to under- or over-treatment. Achieving complete pathologic response in locally advanced disease patients is a protective factor in developing endocrine resistance.

## Figures and Tables

**Table 1.** Demographic characteristics and clinical data of patients, attended the National Cancer Institute - Mexico from January 2014 to December 2016 (N = 200)

Variable	Hormone resistant (n = 65)		Hormone sensitive (n = 135)		*p*-value
	Mean/N	SD/%	Mean/N	SD/%	
Menarche, age (years)	12.48	1.69	12.99	2.01	0.064
Tumor size (cm)	7.15	3.40	5.71	2.68	**0.003**
Ki67 (%)	31.17	23.85	29.59	21.98	0.653
Age <40 years	10	15.4%	55	21	15.8%
Age >50 years	30	46.2%	69	34.5%	0.613
**Body mass index**					
<25 kg/m^2^	18	27.7%	36	26.7%	1.000
>25 kg/m^2^	47	72.3%	99	73.3%	
**Comorbidities**					
Diabetes mellitus	8	12.3%	20	14.8%	0.794
Hypertension	9	13.8%	26	19.3%	0.456
Smoking (+)	15	23.1%	28	20.7%	0.847
Menopause (+)*	31	47.7%	74	54.8%	0.427
**Histological grade**					
Low	10	15.4%	18	13.33%	0.925
Intermediate	27	41.5%	57	42.22%	
High	28	43.1%	60	44.44%	
**Histological type**					
Infiltrating ductal carcinoma	55	84.6%	122	90.4%	0.338
Other	10	15.4%	13	9.6%	
**Immunophenotype**					
Luminal A-like	20	30.8%	44	32.6%	0.479
Luminal B-like	45	69.2%	91	67.4%	
**Clinical stage**					
IA	0	0.0%	5	3.7%	**<0.001**
IIA	1	1.5%	5	3.7%	
IIB	4	6.2%	25	18.5%	
IIIA	15	23.1%	58	43.0%	
IIIB	14	21.5%	33	24.4%	
IIIC	5	7.7%	2	1.5%	
IV	26	40.0%	7	5.2%	
**Clinical outcome**					
Recurrence	37	56.9%	5	3.7%	**<0.001**
Death	20	30.8%	2	1.5%	**<0.001**

SD: Standard deviation.

* Menopause status at diagnosis. Smoking was defined as the reported tobacco usage at some stage in life. Histologic grade was determined from the Scarr-Bloom Richardson scale.

**Table 2.** Clinical stage of breast cancer patients, attended the National Cancer Institute - Mexico from January 2014 to December 2016 (N = 200)

Variable		Hormone resistant (n = 65)		Hormone sensitive (n = 135)		*p*-value
		N	%	N	%	
**T**						
	1	1	1.5%	9	6.7%	**0.004**
	2	9	13.8%	37	27.4%	
	3	27	41.5%	57	42.2%	
	4	28	43.1%	28	20.7%	
	4d	0	0.0%	4	3.0%	
**N**						
	0	5	7.7%	20	14.8%	**<0.001**
	1	17	26.2%	55	40.7%	
	2	30	46.2%	57	42.2%	
	3	13	20.0%	3	2.2%	
**M**						
	0	61	93.8%	131	97.0%	0.488
	1	4	6.2%	4	3.0%	

**Table 3.** Sensitivity analyses for the association between clinical stage and endocrine resistance in breast cancer patients, attended the National Cancer Institute - Mexico from January 2014 to December 2016 (N = 200)

	Unadjusted		Adjusted by age and age at menarche		Adjusted by age, age at menarche, and Ki67		Adjusted by age, age at menarche, Ki67, phenotype (luminal A vs. B), BMI, histological subtype (Ductal vs. Non-ductal)	
	RR	*p*-value	RR	*p*-value	RR	*p*-value	RR	*p*-value
	95% CI		95% CI		95% CI		95% CI	
Clinical stage^a^	4.20	**0.004**	4.14	**0.006**	4.11	**0.006**	4.39	**0.004**
	1.560, 11.302		1.527,11.246		1.520,11.280		1.502,11.427	
AIC	245.84		245.67		247.63		252.86	

95% CI: 95% confidence interval. BMI: Body mass index. SBR: Scarff-Bloom-Richardson.

a: Included as continuous variable.

b: Included as categorical variable.

A: Dichotomized (I-IIB vs. IIIA-IV). AIC: Akaike information criteria

**Table 4.** Multivariable-adjusted Cox regression model for the association between endocrine resistance and mortality in breast cancer women, attended the National Cancer Institute in Mexico City from January 2014 to December 2016 (N = 200)

	HR	95% CI	*p*-value
Endocrine resistance	23.7	5.20, 108.42	**<0.001**

**Table 5.** Sensitivity analyses for the role of complete pathologic response towards the association between clinical stage and endocrine resistance in locally advanced breast cancer patients, attended the National Cancer Institute - Mexico from January 2014 to December 2016 (N = 200)

	RR	*p*-value	RR	
	95% CI		95% CI	*p*-value
Clinical stage^a^	2.74	**0.021**	2.92	**0.018**
	1.17, 6.42		1.20, 7.11	
Complete pathologic response			0.15	**0.020**
			0.03, 0.75	
AIC	158.79		153.26	

95% CI: 95% confidence interval. Adjusted by age, age at menarche, Ki67, phenotype (luminal A vs. B), BMI, histological subtype (Ductal vs. Non-ductal), and histological grade (low vs intermediate + high). BMI: Body mass index.

a: Dichotomized (I-IIB vs. IIIA-IV). AIC: Akaike information criteria

## Data Availability

The datasets generated and/or analyzed during the current study are not publicly available due to hospital policy but are available from the corresponding author on reasonable request.

## Abbreviations

BC: breast cancer
ER: Estrogen Receptor
HR: Hazard Ratio
TCGA: The Cancer Genome Atlas Project
SERMs: Selective Endocrine Resistance Modulators
SERDs: Selective Endocrine Resistance Down-regulators
AIs: Aromatase Inhibitors
CDK4: Cyclin-Dependent Kinases 4
CDK6: Cyclin-Dependent Kinases 6
EBCTCG: Early Breast Cancer Trialists Collaborative Group
ABC2: Advance Breast Cancer 2
HER2: Epidermal Growth Factor Receptor 2
CRP: Complete Pathological Response
ASCO: American Society of Clinical Oncology
CAP: College of American Pathologists
AJCC: American Joint Committee on Cancer
AIC: Akaike Information Criteria
EBCTCG: Early Breast Cancer Trialist Collaborative Group
